# Hydroxypropyl-β-cyclodextrin Enhances Oral Absorption of Silymarin Nanoparticles Prepared Using PureNano™ Continuous Crystallizer

**DOI:** 10.3390/pharmaceutics14020394

**Published:** 2022-02-10

**Authors:** Risako Onodera, Tomohiro Hayashi, Keiichi Motoyama, Kohei Tahara, Hirofumi Takeuchi

**Affiliations:** 1 Laboratory of Pharmaceutical Engineering, Gifu Pharmaceutical University, 1-25-4 Daigaku-Nishi, Gifu 501-1196, Japan; deragon@kumamoto-u.ac.jp (R.O.); hayashi.tomohiro@ma.mt-pharma.co.jp (T.H.); tahara@gifu-pu.ac.jp (K.T.); 2 Graduate School of Pharmaceutical Sciences, Kumamoto University, 5-1 Oe-honmachi, Chuo-ku, Kumamoto 862-0973, Japan; motoyama@kumamoto-u.ac.jp

**Keywords:** silymarin, hydroxypropyl-β-cyclodextrin, continuous crystallizer (PureNano^TM^), crystallization, oral bioavailability

## Abstract

The oral bioavailability of drugs is limited by factors such as poor membrane permeability, low solubility, and low dissolution rate. Silymarin (SLM) is a health-food active ingredient that is good for immunosuppression and tumor suppression. However, obtaining a good oral bioavailability is difficult owing to its poor solubility and low dissolution ability. To overcome these concerns, we previously prepared SLM nanoparticles (NPs) using the high-pressure crystallization method (PureNano^TM^) and freeze-dried them with erythritol (Ery) or hydroxypropyl-β-CyD (HP-β-CyD) as a water-soluble dispersion stabilizer. In the present study, we investigated the mechanism underlying the improved absorption of SLM/hypromellose (HPMC)/HP-β-CyD NPs after oral administration. The SLM/HPMC nano-suspension prepared using PureNano^TM^ exhibited a narrow size distribution. The size of the SLM/HPMC/HP-β-CyD NPs was approximately 250 nm after hydration. The SLM/HPMC/HP-β-CyD NPs were rapidly dissolved, and demonstrated a high solubility under supersaturated conditions. Additionally, they exhibited good wettability and their membrane permeability was improved compared with that of SLM original powder. These results suggest that the formulation of SLM NPs using PureNano^TM^ and freeze-drying with HP-β-CyD improves the absorption of SLM after oral administration by enhancing solubility, wettability, and membrane permeability.

## 1. Introduction

The oral bioavailability of a drug is limited by factors, such as membrane permeability, solubility, and dissolution rate [1,2]. In general, miniaturization of compound crystals and solid dispersions are known to improve the solubility of poorly water-soluble compounds [3,4,5,6] and is advantageous by increasing dissolution rate due to increased surface area. According to the Noyes-Whitney equation, an increase in the surface area results in fast rates of drug dissolution [7]. For poorly water-soluble drugs, such as sirolimus [8], aprepitant [9], and fenofibrate [10], micronization technology has been applied to improve the solubility of compounds. There are two types of miniaturization methods: top-down [11,12,13] and the bottom-up. The bottom-up method can produce nano-sized particles by crystallization from a solution or coacervation with a sharp size distribution. We have previously prepared nanocrystal suspensions of poorly water-soluble drugs using a continuous crystallizer (PureNano^TM^) [14]. In the PureNano^TM^ system, crystallization is immediately performed by a high shearing force, which is generated after the rapid feeding of aqueous and organic solutions to the collision jet field. Therefore, well-size-controlled particles can be prepared using PureNano^TM^, compared with the usual crystallization method. However, nanoparticles gradually aggregate and induce crystal growth because of the high surface energy of nanoparticles, which is concerning [15]. To overcome these concerns, a dispersion stabilizer is necessary to avoid particle aggregation and crystal growth.

Silymarin (SLM) is an ingredient extracted from milk thistle [16], which contains the most effective silybin A and silybin B, as well as is silydianin, silychristin, taxifolin, and quercetin [17,18]. SLM has beneficial pharmacological activities such as immunosuppressive action, antitumor action [16], and hepatoprotective action [19]. However, SLM is poorly water-soluble, which limits its absorption after oral administration. Methods to improve the absorption of SLM after oral administration have been reported, including micronization, such as nanocrystals, nanosuspensions, and solid dispersions, and the use of carriers, such as cyclodextrins, liposomes, and lipid nanoparticles [20,21,22,23]. It has been suggested that improving the solubility of SLM in either technique improves the absorbability after oral administration. However, there are few reports on the effect of improving the solubility of SLM using both miniaturization and carriers.

Cyclodextrins (CyDs) are cyclic oligosaccharides consisting of six to eight glucose units linked by α-1,4-glycosidic linkages. The potential use of natural CyDs and their synthetic derivatives has been extensively studied to improve certain properties of drugs, such as solubility, stability, and bioavailability. One of the most useful applications of CyDs in dosage form design is to enhance the solubility of poorly water-soluble drugs by complex formation [24,25]. Among them, β-CyD is widely used as a pharmaceutical additive for inclusion, but its low solubility may be a problem. Therefore, water-soluble CyD derivatives have been developed to improve their functionality. Hydroxypropyl-β-CyD (HP-β-CyD), a hydroxypropylated product, is used as a solubilizer for oral syrups and injections of itraconazole [26]. Recently, it has been reported that CyD is utilized as additives to prepare the submicron-sized drug particles by supercritical treatment or co-milling [27,28].

We previously reported the preparation of SLM nanoparticles by PureNano^TM^ using HP-β-CyD or erythritol (Ery) as additives in the freeze-drying process [29]. The SLM/HP-β-CyD nanoparticles exhibited 3.3-fold and 4.2-fold increase in AUC_0–6_ h and Cmax, respectively, compared to SLM/Ery nanoparticles. However, the mechanism for improving the absorbability of SLM/HP-β-CyD nanoparticles has not been investigated in detail.

Based on this background, in this study, we investigated the relationship of the enhanced absorption of SLM to crystallization by PureNano^TM^ and improved its solubility by the adding HP-β-CyD. We evaluated the physicochemical properties, dissolution rates, and membrane permeabilities of various SLM nanoparticles.

## 2. Materials and Methods

### 2.1. Materials

SLM and Hypromellose (HPMC) were donated by the FANCL Corporation (Kanagawa, Japan) and Shin-Etsu Chemical Corporation (Tokyo, Japan), respectively. Mannitol and trehalose dihydrate were purchased from Merck (Darmstadt, Germany) and FUJIFILM Wako Pure Chemical (Osaka, Japan), respectively. Sugar ester (S-1670) and Ery were purchased from Mitsubishi-Kagaku Foods Corporation (Tokyo, Japan). α-CyD and HP-β-CyD were purchased from Nacalai Tesque, Inc. (Kyoto, Japan) and Junsei Chemical (Tokyo, Japan), respectively. Dulbecco’s modified Eagle medium and fetal bovine serum were purchased from Nissui Pharmaceutical Co., Ltd. (Tokyo, Japan) and Nichirei (Tokyo, Japan), respectively. All other chemicals and solvents were of analytical reagent grade, and deionized double-distilled water was used throughout the study.

### 2.2. Cell Culture

Caco-2 cells, a human colon adenocarcinoma cell line, were cultured as previously reported [15]. Transepithelial/transendothelial electrical resistance values of Caco-2 cell monolayers grown on Transwell^®^ (12-well, Corning, NY, USA) were measured using a Millicel^®^-ERS voltmeter (Millipore, MA, USA) and was reported to be 808 ± 7.8 Ω/cm^2^.

### 2.3. Preparation of SLM Nanoparticles (NPs)

#### 2.3.1. Preparation of SLM NPs Using PureNano^TM^

SLM NPs were prepared as reported previously [15]. Briefly, SLM (1 g), HPMC (0.5% *w/v*), or S-1670 (0.5% *w/v*) were dissolved in ethanol (20 mL) and distilled water (200 mL), respectively. Subsequently, the NP suspension was prepared using PureNano^TM^ (Microfluidics, Westwood, MA, USA) and evaporated for 6 h to remove the ethanol. After freeze-drying for 24 h, fine nanoparticles were obtained.

#### 2.3.2. Drug Content of SLM NPs

The quantities of SLM NPs equivalent to 33% of SLM were dissolved in a water/dimethyl sulfoxide (DMSO) solution (1:1, *v/v*). The SLM content was analyzed using high-performance liquid chromatography (HPLC, JASCO Corp., Tokyo, Japan). The measurement conditions were as follows: a Jasco HPLC system (Tokyo, Japan) and a UV detector at 288 nm (Tokyo, Japan); a Phenomenex Luna 5u C18 (2) column (4.6 × 250 mm; Phenomenex, Torrance, CA, USA); mobile phase of trichloroacetic acid (pH 2.5)/ methanol (71:20 *v/v* at 0–20 min, 72:29 to 35:65 *v/v* at 20–40 min); flow rate of 1.0 mL/min; column temperature of 30 °C.

### 2.4. Physicochemical Properties of SLM NPs

#### 2.4.1. Particle Size and ζ-Potential Analysis

Briefly, 0.1% (*w/v*) SLM NP suspension in water was prepared. After ultrasonication for 2 min, the particle size and ζ-potential of the SLM NP suspension were measured using a ZetaSizer Nano ZS (Malvern, Worcestershire, UK).

#### 2.4.2. X-ray Diffraction Study

Powder X-ray diffraction patterns were measured using a Bruker D8 Advance X-ray diffractometer (Karlsruhe, Germany) under the following conditions: Ni-filtered Cu-Kα radiation (1.5406 Å), 40 kV, 40 mA, and over the 2θ rage 5–30°.

#### 2.4.3. Scanning Electron Microscopy (SEM)

The morphology analysis of the SLM nanoparticles was performed using SEM, as reported previously. SLM nanoparticles were sputtered with gold (5 min, 6–8 mA) using an Auto Fine Coater JFC-1600 (JEOL Ltd., Tokyo, Japan) and observed with a JSM-6510LV (JEOL Ltd., Tokyo, Japan) at an accelerating voltage of 8–15 kV.

### 2.5. In Vitro Release Test

The release test of SLM nanoparticles (equivalent to 3.6 mg) was performed under sink conditions according to the Japanese Pharmacopoeia (18th Edition), with 900 mL of distilled water at 37 °C ± 0.5 °C, and stirring at a rotation speed of 100 rpm. At appropriate intervals, an aliquot (1 mL) of the dissolution medium was withdrawn using a 0.2 μm polytetrafluoroethylene (PTFE) filter, and then diluted using DMSO. The content of silybin in the release medium was HPLC (JASCO Corp., Tokyo, Japan).

### 2.6. Membrane Permeability Studies

Caco-2 cells were cultured on tissue-culture-treated polycarbonate Transwell^®^ for 21 days before use. Caco-2 cell monolayers were washed thrice with HBSS-HEPES buffer (10 mM, pH 7.4) and incubated in HBSS-MES buffer (10 mM, pH 6.0) containing SLM NPs for 180 min. The concentration of SLM in the basolateral chamber was determined using HPLC.

### 2.7. Contact Angle

Briefly, various tablets (diameter 8 mm) were prepared from SLM NPs using a universal tensile compression tester (AUTOGRAPH, Shimadzu Corp., Kyoto, Japan) under a compression pressure of 200 MPa and a compression rate of 10 mm/min. The contact angles of the various SLM NPs were determined using a contact angle meter (DM100, Kyowa Interface Science, Saitama, Japan).

### 2.8. Solubility

Various samples (10 mg) were suspended in water (30 mL) and stirred at 100 rpm overnight. One milliliter of the collected sample solution was subjected to ultracentrifugation at 75,000 rpm for 12 min at 4 °C. Thereafter, the supernatant was filtered through a 0.2 μm hydrophilic PTFE filter and diluted with DMSO before HPLC analysis. HPLC conditions for the determination of SLM in the supernatant were the same as those used for the in vitro release test.

### 2.9. Phase Solubility

The inclusion ability of SLM with Ery, α-CyD and HP-β-CyD were evaluated by the solubility method. Ery, α-CyD and HP-β-CyD solutions (0–25 mM) were prepared. SLM (125 mg) was into vial tubes, Ery or CyDs solutions (1 mL) of various concentrations were added, and equilibration was performed at room temperature with constant shaking. After equilibration, the vials were centrifuged and filtered through a 0.2 μm hydrophilic PTFE filter. The amount of silymarin in the filtrate was quantified by HPLC. HPLC conditions for the determination of SLM were the same as those used for the drug content test.

### 2.10. Data Analysis

Data are presented as the mean ± S.E. Statistical significance of mean coefficients for the studies was performed by analysis of variance followed by Scheffe’s test. The *p*-values for significance were set at 0.05.

## 3. Results and Discussion

### 3.1. Preparation of SLM Nanoparticles (NPs)

#### 3.1.1. Preparation of SLM NPs Using PureNano^TM^

SLM was dissolved in ethanol and distilled water and placed in PureNano^TM^ to prepare an SLM suspension. The particle size of the SLM suspension was measured immediately. The organic solvent in the SLM suspension was distilled off using a rotary evaporator, and particle size was measured. The suspension was powdered by freeze-drying (FD) to obtain SLM NPs. The redispersibility of the SLM NPs (FD product) was evaluated by measuring the particle size after redispersion in distilled water. Table 1 shows the particle size and polydispersity index (PDI) of the SLM suspensions after PureNano^TM^ or evaporation of the organic solvent, and SLM NPs after FD. The particle size and PDI of SLM suspensions just after nano-crystallization by PureNano^TM^ was 188.6 nm and 0.138, respectively. Meanwhile, the particle size of the SLM suspension after evaporation was slightly increased due to Ostwald ripening, because PDI of the nanosuspension did not change [30,31]. Moreover, the size of SLM NPs after FD was more than 1000 nm, probably due to agglomeration, suggesting poor redispersibility in water (Table 1). These data indicate the importance of water-soluble dispersion stabilizers during the FD process to obtain SLM NPs with high redispersibility.

#### 3.1.2. Effect of Dispersion Stabilizer on Preparation of SLM NPs

SLM NPs, SLM/HPMC, and SLM/S-1670 with sub-micron sizes and narrow size distributions were successfully formulated. After crystallization, particle size of the SLM NPs was approximately 300 nm, regardless of the type of additive. In contrast, the particle size of SLM/HPMC NPs after FD was increased to 677.7 nm, but smaller than that of SLM/S-1670 NPs after FD (Table 2). This might be because the weight ratio of HPMC was higher than that of S-1670, due to steric hindrance through aggregation. Moreover, the particle size of SLM/HPMC NPs after one week was slightly increased; up to 697.1 nm. In contrast, SLM/S-1670 NPs aggregated after one week, and their particle size was more than 1000 nm. Because of the interparticle interaction in SLM/S-1670 NPs was stronger than that in SLM/HPMC NPs (HLB: hydrophilic-lipophilic balance value of S-1670 was 16). Therefore, we selected HPMC as a dispersion stabilizer rather than S-1670 in subsequent experiments.

Next, to examine the effect of HPMC concentration on particle size of SLM/HPMC NPs, we prepared SLM/HPMC NPs with various HPMC concentration (0.5–3%, *w/w*) (Table 3). The particle size of SLM/HPMC NPs increased as the HPMC content increased. Van den Mooter et al. have reported that HPMC adsorbs to the crystal surface of a drug and forms intramolecular hydrophobic interactions via the hydrophilic and hydrophobic groups of HPMC [32,33]. This is in agreement with the findings of Kayaert et al. [32,33]. Therefore, it is presumed that the increase in the particle size of the SLM/HPMC suspensions depending on the HPMC concentration was likely due to the adsorption of HPMC on the surface of silymarin and the formation of HPMC layers. Moreover, it was clarified that the particle size after FD maintained the nanoparticles only when the weight ratio of SLM to HPMC was 1:1. In contrast, particle agglutination was observed at weight ratios of SLM to HPMC of 1:0.5 and 1:3. This could be because when the HPMC concentration was low, it was presumed that the amount of HPMC adsorbed on the SLM fine particles was insufficient. The SLM/HPMC suspension was concentrated during the FD process, and contact between the particles could not be suppressed. In contrast, when the HPMC concentration was high, the viscosity of the SLM/HPMC suspension increased and the molecular motility of the particles was suppressed, resulting in agglomeration of the particles. In addition, HPMC was considered to adsorb on SLM caused an interaction between particles and aggregation. These results indicate that selecting the optimal concentration of HPMC due to the preparation of SLM/HPMC nanoparticles is important.

#### 3.1.3. Effect of Cryoprotectants on Preparation of SLM NPs

In general, it is known that particles aggregate into coarse particles during the drying process. Therefore, cryoprotectants have been used to suppress particle aggregation [34,35]. Next, we evaluated the effect of various cryoprotectants on the aggregation of FD powder of SLM/HPMC NPs. As shown in Table 4, the particle sizes of SLM/HPMC in the presence of cryoprotectants such as erythritol (Ery), mannitol, trehalose, α-CyD, and HP-β-CyD were smaller than those without cryoprotectants (particle size: 617 nm, Table 3), suggesting that each cryoprotectant suppressed the aggregation of SLM/HPMC NPs. It is assumed that these sugar cryoprotectants dissolve in water and suppress aggregation by interposing between the SLM/HPMC NPs. These results suggest that sugar structures have little effect on the suppression of aggregation. In addition, SLM contents in the SLM/HPMC/Ery NP, SLM/HPMC/α-CyD NP and SLM/HPMC/HP-β-CyD NP was 32.25 ± 0.01%, 31.30 ± 0.64% and 31.85 ± 0.41%, respectively, and were close to the theoretical SLM content of 33.3% (Table 5). These results imply that SLM does not disappear or decompose during particle preparation. However, in drug formulation, it is important to assess the physical and chemical stability of the formulation. Further elaborate study on nanoparticle stability is necessary.

### 3.2. Physicochemical Properties of SLM NPs

#### 3.2.1. Crystalline State Analysis

**Figure 1:**
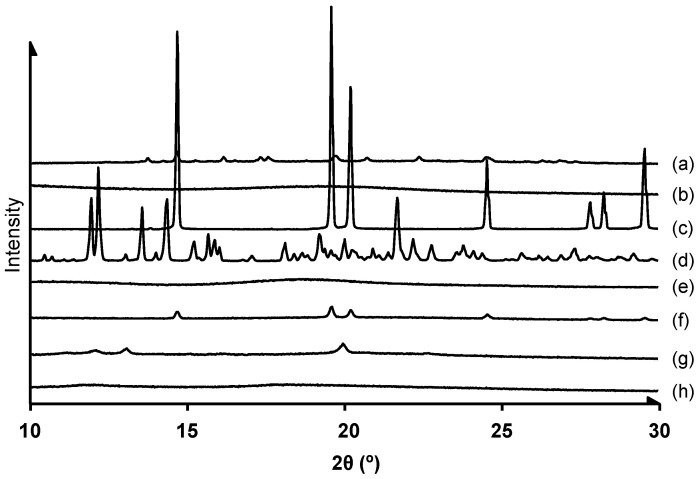
X-ray diffraction patterns of various samples (a) Silymarin (SLM) original, (b) hypromellose (HPMC) original, (c) erythritol (Ery) original, (d) α-cyclodextrin (α-CyD) original, (e) hydroxypropyl (HP)-β-CyD original, (f) SLM/HPMC/Ery NPs (1/1/1), (g) SLM/HPMC/α-CyD NPs (1/1/1), (h) SLM/HPMC/HP-β-CyD NPs (1/1/1). The weight ratio of SLM/HPMC/stabilizers was 1/1/1.

We performed powder X-ray diffraction analysis of the FD powder of SLM/HPMC NPs (Figure 1). Powder X-ray diffraction patterns of SLM, Ery, and α-CyD showed many peaks, suggesting their crystalline forms in solid state. On the other hand, those of HPMC and HP-β-CyD were a halo, indicating that they were amorphous. In addition, the X-ray diffraction patterns of SLM/HPMC/Ery physical mixture (PM), SLM/HPMC/α-CyD PM and SLM/HPMC/HP-β-CyD PM showed silymarin-derived peaks, suggesting their crystalline forms (Supplemental Figure S1A–C). On the other hand, the diffraction peaks derived from Ery decreased in the SLM/HPMC/Ery NPs suggesting that the high-pressure crystallization treatment reduced the crystallinity of Ery. Moreover, the X-ray diffraction pattern of SLM/HPMC/HP-β-CyD NPs was halo, indicating that they were amorphous. It is reported that HP-β-CyD forms inclusion complexes with drugs, resulting in the formation of amorphous particles [36]. Therefore, it is suggested that SLM/HPMC/HP-β-CyD NPs formed amorphous nanoparticles due to the formation of inclusion complex with SLM and CyD during the high-pressure crystallization procedure. On the other hand, new peaks appeared in the SLM/HPMC/α-CyD NPs. We confirmed that SLM/α-CyD NPs showed similar X-ray diffraction peaks to those of SLM/HPMC/α-CyD NPs, suggesting that the new peaks originated from the inclusion complex of SLM and α-CyD (Supplemental Figure S1B).

#### 3.2.2. Scanning Electron Microscopy (SEM) of SLM NPs

**Figure 2:**
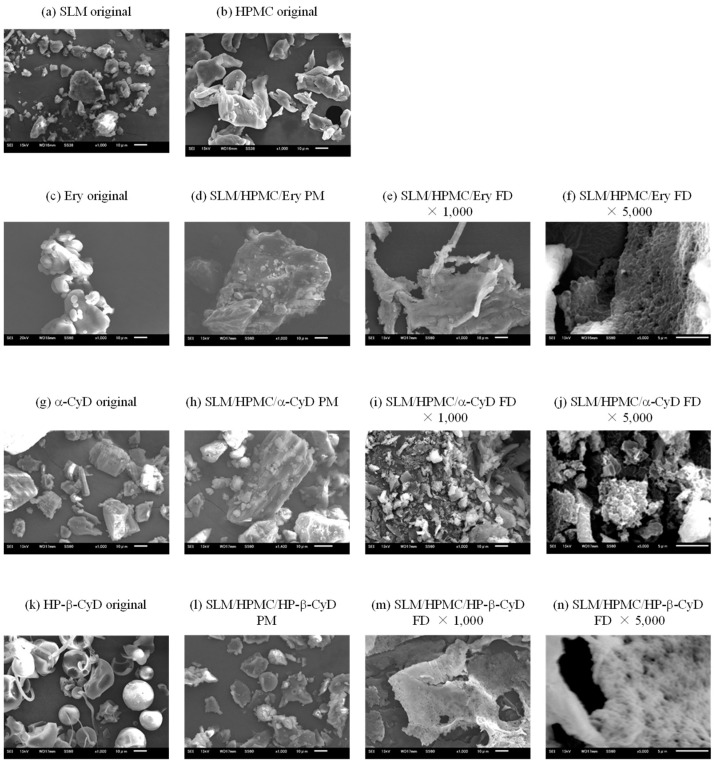
Morphological observation of silymarin (SLM), hypromellose (HPMC), erythritol (Ery) and SLM/HPMC/Ery nanoparticles (NPs). The weight ratio of SLM/HPMC/stabilizers was 1/1/1. The experiments were performed independently thrice, and representative images are shown. X-ray diffraction patterns of various samples. (**a**) Silymarin (SLM) original, (**b**) hypromellose (HPMC) original, (**c**) erythritol (Ery) original, (**d**) SLM/HPMC/Ery physical mixture (PM), (**e**,**f**) SLM/HPMC/Ery freeze dry (FD), (**g**) α-cyclodextrin (α-CyD) original, (**h**) SLM/HPMC/α-CyD PM, (**i**,**j**) SLM/HPMC/α-CyD FD, (**k**) hydroxypropyl (HP)-β-CyD original, (**l**) SLM/HPMC/HP-β-CyD PM, (**m**,**n**) SLM/HPMC/HP-β-CyD FD. The weight ratio of SLM/HPMC/stabilizers was 1/1/1.

Representative photographs of the SLM original powder, the physical mixture method, and the FD method are shown in Figure 2. The original SLM powder was amorphous and angular particles. In addition, particles of the SLM original powder were coarse (5–20 μm) (Figure 2a). In contrast, particle shapes of HPMC and Ery were found to be rounded (Figure 2b,c). The original powders of SLM, HPMC, and Ery were adsorbed on each other in the physical mixture of SLM/HPMC/Ery (Figure 2d). The FD powder of SLM/HPMC/Ery NPs showed different particle shapes from the original powders of SLM, HPMC, or Ery (Figure 2e). α-CyD and HP-β-CyD original powders showed angular and spherical shapes, respectively (Figure 2g,k). The original powders of SLM, HPMC, and CyDs were adsorbed to each other, and the SLM original powder, HPMC, and CyDs were adsorbed to each other in the physical mixture of SLM/HPMC/CyDs (Figure 2h,l). On the other hand, the SLM/HPMC/α-CyD NPs and SLM/HPMC/HP-β-CyD NPs showed completely different particle shapes (Figure 2i,m). Interestingly, the SLM/HPMC/α-CyD NPs revealed an angular shape and provided a new crystal form (Figure 2j), probably due to interaction between SLM and α-CyD. SLM/HPMC/HP-β-CyD NPs demonstrated irregularities on the particle surface (Figure 2n), similar to SLM/HPMC/Ery NPs (Figure 2f).

### 3.3. In Vitro Release Test

**Figure 3:**
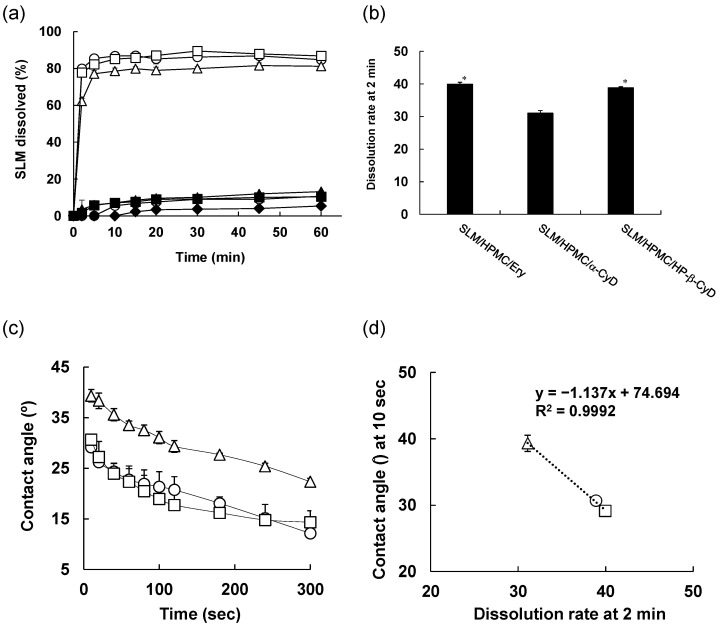
Dissolution behavior (**a**,**b**), Contact angle (**c**) and Correlative Evaluation (**d**) of various silymarin/hypromellose nanoparticles (SLM/HPMC NPs). ◆: SLM original, ●: SLM/HPMC/erythritol (Ery) PM (1/1/1), ▲: SLM/HPMC/α-cyclodextrin(α-CyD) PM, ■: SLM/HPMC/HP-β-CyD PM, 〇: SLM/HPMC/Ery NPs, △: SLM/HPMC/α-CyD NPs, □: SLM/HPMC/HP-β-CyD NPs. The weight ratio of SLM/HPMC/stabilizers was 1/1/1. (**a**) Dissolution behavior up to 60 min. (**b**) Dissolution behavior up to 2 min. * *p* < 0.05, compared with SLM/HPMC/α-CyD NPs. (**c**) Contact angles of SLM/HPMC/Ery NPs, SLM/HPMC/α-CyD NPs, and SLM/HPMC/HP-β-CyD NPs. (**d**) Correlative evaluation between dissolution rate and contact angle of SLM/HPMC/Ery NPs, SLM/HPMC/α-CyD NPs, and SLM/HPMC/HP-β-CyD NPs. Data represent the mean ± SE of three experiments.

Refining the SLM original powder increased the specific surface area. In addition, it has been reported that reducing the particle size of a poorly water-soluble drug improves its wettability to water [37]. Therefore, we evaluated the release properties of SLM NPs. Figure 3 shows the in vitro release test of SLM original powder, physical mixtures, or NP formulations of SLM/HPMC/Ery, SLM/HPMC/α-CyD, and SLM/HPMC/HP-β-CyD. SLM released from the original powder after 60 min was approximately 5% (Figure 3a). The amount of SLM released from the physical mixtures of SLM/HPMC/Ery, SLM/HPMC/α-CyD, and SLM/HPMC/HP-β-CyD were slightly higher than those from the SLM original powder (Figure 3a). SLM/HPMC/Ery NPs, SLM/HPMC/α-CyD NPs, and SLM/HPMC/HP-β-CyD NPs showed drastically high SLM release from their NPs (almost 80% SLM release), probably due to the enhancement of wettability by miniaturization. In contrast, the SLM release rate in SLM/HPMC/α-CyD NPs at 2 min was slightly lower than that in SLM/HPMC/Ery NPs or SLM/HPMC/HP-β-CyD NPs (Figure 3b), as SLM/HPMC/α-CyD NPs showed angular particle shapes different from those of the other NP formulations (Figure 2).

To evaluate the wettability of various SLM NPs, the contact angles of SLM/HPMC/Ery NPs, SLM/HPMC/α-CyD NPs, and SLM/HPMC/HP-β-CyD NPs were measured (Figure 3c). Various SLM NPs were compression-molded at 200 MPa to prepare the pellets. Distilled water was added dropwise to the pellets, and the contact angle was evaluated for 300 s. The contact angle of each SLM NP decreased in a time-dependent manner. In contrast, the contact angle of SLM/HPMC/α-CyD NPs was higher than that of SLM/HPMC/Ery NPs or SLM/HPMC/HP-β-CyD NPs. These results indicate that the wettability of SLM/HPMC/α-CyD NPs was lower than that of SLM/HPMC/Ery NPs or SLM/HPMC/HP-β-CyD NPs. Notably, there was a good correlation between the contact angle at 10 s and the release rate up to 2 min (Figure 3d). Taken together, these results suggest that the wettability of the SLM NPs is involved in the initial elution rate.

### 3.4. Solubility Study of SLM NPs

**Figure 4:**
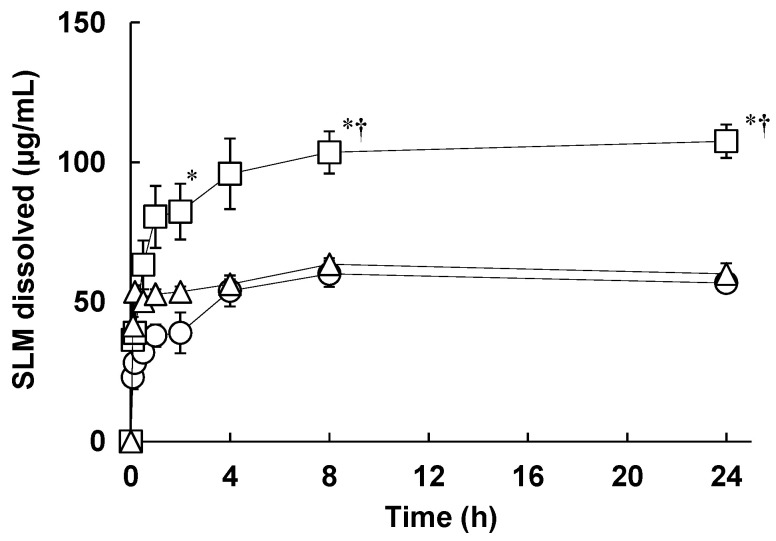
Dissolution behavior of silymarin/Hypromellose/erythritol nanoparticles (SLM/HPMC/Ery) NPs, SLM/HPMC/α-cyclodextrin (α-CyD) NPs, and SLM/HPMC/HP-β-CyD NPs. 〇: SLM/HPMC/Ery NPs, △: SLM/HPMC/α-CyD NPs, □: SLM/HPMC/HP-β-CyD NPs. The weight ratio of SLM/HPMC/stabilizers was 1/1/1. Data represent the mean ± SE of three experiments. * p < 0.05, compared to SLM/HPMC/Ery NPs. † *p* < 0.05, compared with SLM/HPMC/α-CyD NPs.

The potential use of CyDs has been extensively studied to improve the solubility and bioavailability of drugs through the formation of inclusion complexes. Next, we evaluated the solubility of the SLM NPs containing CyDs (Figure 4). Briefly, various SLM NPs were suspended in water and stirred at 100 rpm for 24 h. SLM concentration in water at 24 h was determined as the solubility of SLM, as the solution equilibria were achieved at 24 h. As a result, the solubility of SLM/HPMC/Ery NPs and SLM/HPMC/α-CyD NPs were 57 ± 3.0 μg/mL and 60 ± 3.8 μg/mL, respectively. On the other hand, the solubility of SLM/HPMC/HP-β-CyD NPs was significantly enhanced (108 ± 6.0 μg/mL, compared to that of SLM/HPMC/Ery NPs or SLM/HPMC/α-CyD NPs (Figure 4)). In addition, we investigated whether HP-β-CyD was able to form inclusion complex with SLM by solubility method. The solubility phase diagram is classified into A-type, which indicates a soluble complex, and B-type, which indicates poor water solubility. In addition, A-type has A_L_ (liner diagram), A_p_ (positive deviation from linearity), A_N_ (negative deviation from linearity), and B-type has B_S_ (the complex has some but limited solubility) and B_I_ (the complex is insoluble) [38]. The Ery and α-CyD showed little solubilizing effect on silymarin. On the other hand, HP-β-CyD showed A_L_-type solubility phase diagram in which the solubility of SLM increased linearly with increasing HP-β-CyD concentration, suggesting the formation of a soluble inclusion complex. In addition, the A_L_-type phase diagram suggests that the drug and CyD form an apparent 1:1 inclusion complex. Moreover, the stability constants of SLM/Ery and SLM/α-CyD calculated from Higuchi and Connor’ equation were 1.1 × 10^1^ M^−1^ and 2.3 × 10^1^ M^−1^, respectively, whereas the stability constant of SLM/HP-β-CyD was as high as 7.1 × 10^3^ M^−1^. These results suggest that HP-β-CyD could enhance the solubility of SLM by forming an inclusion complex.

### 3.5. Membrane Permeability Test of SLM NPs

**Figure 5:**
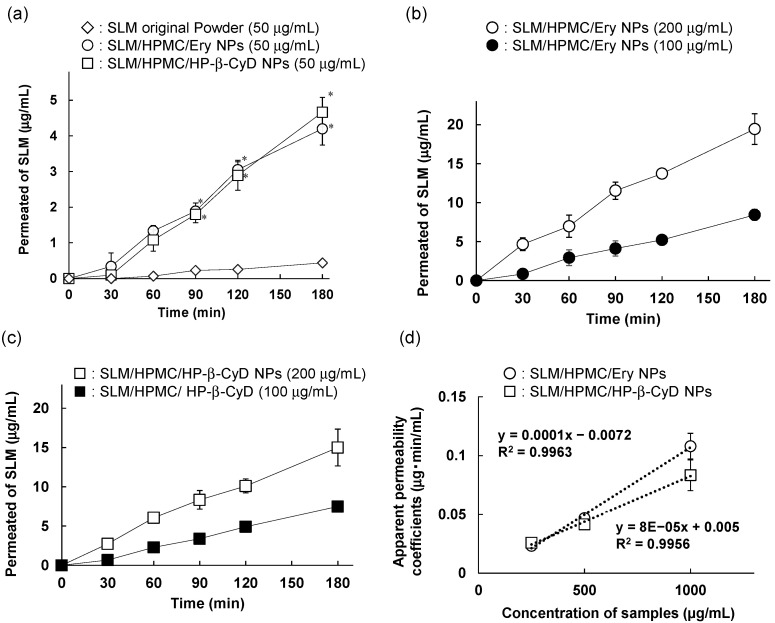
Membrane permeability of silymarin (SLM) original powder, si-lymarin/hypromellose/erythritol nanoparticles (SLM/HPMC/Ery NPs) and SLM/HPMC/HP-β-CyD NPs against Caco-2 cell monolayers. (**a**) Membrane permeability assay of SLM original powder, SLM/HPMC/Ery NPs or SLM/HPMC/HP-β-CyD NPs at the SLM concentration of 50 mg/mL. * *p* < 0.05, compared with SLM original powder. (**b**,**c**) Membrane permeability of SLM/HPMC/Ery NPs and SLM/HPMC/HP-β-CyD NPs at the SLM concentration of 100 mg/mL or 200 mg/mL. (**d**) Correlative evaluation between SLM concentrations of NPs and apparent permeability coefficients. Data represent the mean ± S.E. of 3–4 experiments.

The membrane permeability of a drug is an important parameter for estimating the bioavailability after administration. Therefore, we next evaluated the membrane permeability of SLM released from SLM/HPMC/Ery NPs and SLM/HPMC/HP-β-CyD NPs in CaCo-2 cell monolayers. Poor membrane permeability was observed in the original SLM powder (Figure 5a). In contrast, the time-dependent membrane permeability of SLM in SLM/HPMC/Ery NPs or SLM/HPMC/HP-β-CyD NPs was observed (Figure 5a). In addition, the membrane permeability of SLM in SLM/HPMC/Ery NPs or SLM/HPMC/HP-β-CyD NPs increased as the SLM content in the formulation was increased up to 200 μg/mL (Figuere 5b,c). We confirmed no significant change in the membrane resistance value under these experimental conditions (data not shown), indicating that the membrane permeation of SLM was caused by passive diffusion rather than paracellular route due to the opening of the tight junction. A linear correlation between the apparent permeability coefficients and SLM concentrations in NP formulations was observed in SLM/HPMC/Ery NPs or SLM/HPMC/HP-β-CyD NPs (Figure 5d). Linearity between SLM/HPMC/Ery NPs and SLM/HPMC/HP-β-CyD NPs were slightly different (Figure 5d). CyD is known to improve the apparent molecular weight of drugs through the formation of inclusion complexes, resulting in lowering of membrane permeability of the drug, even though CyDs can enhance drug solubility in the solution [39,40]. Therefore, the membrane permeability of SLM in SLM/HPMC/HP-β-CyD NPs decreased as the CyD concentration increased, compared with that of SLM/HPMC/Ery NPs. However, in our previous study, SLM/HPMC/HP-β-CyD NPs significantly enhanced the bioavailability of SLM after oral administration to rats compared with SLM/NPMC/Ery NPs [29], and the in vitro membrane permeability of SLM/HPMC/HP-β-CyD NPs was slightly lower than that of SLM/NPMC/Ery NPs. Taken together, these results suggest that HP-β-CyD enhanced bioavailability of SLM in the SLM/HPMC NPs prepared by PureNano^TM^, probably because of enhancement of passive diffusion of SLM by the solubility enhancing effect.

## 4. Conclusions

To improve the dissolution rate and solubility of SLM, a poorly water-soluble drug, we developed an SLM/HPMC/HP-β-CyD NP formulation. We revealed that HP-β-CyD was a potential cryoprotectant in NP formulation using an SLM nano-suspension prepared by PureNano^TM^. SLM/HPMC/HP-β-CyD NPs showed good redispersion ability and rapid dissolution rate, probably due to the complex formation with SLM. In this formulation, HP-β-CyD enhanced drug solubility and wettability, resulting in the in vivo absorption of SLM compared with SLM/HPMC/Ery NPs and SLM/HPMC/α-CyD NPs as reported previously. However, in improving the absorption of drugs after oral administration, it is important to evaluate not only solubility and membrane permeability, but also the phase II metabolism and excretion in bile. Hence, the effect of nanoparticles on the enhancement of silymarin absorption needs to be further investigated.

## Figures and Tables

**Table 1:** Particle sizes of silymarin (SLM) suspensions and freeze-dried (FD) powder of SLM.

Condition	Particle Size (nm)	PDI
PureNano^TM^	188.6	0.138
Evaporation	224.8	0.166
Freeze Dry (FD)	>1000	Not detected

**Table 2:** Effects of hypromellose (HPMC) and S-1670 on particle sizes of suspension prepared by PureNano^TM^, evaporated samples, freeze drying samples (FD) and FD samples stored for one week.

Compound	Stabilizer	Condition	Particle Size (nm)	PDI
		PureNano^TM^	337.6	0.045
	HPMC	Evaporation	339.0	0.165
		FD	677.7	0.463
SLM		FD after 1 week	697.1	0.478
		PureNano^TM^	281.5	0.115
	S-1670	Evaporation	329.5	0.221
		FD	912.1	0.496
		FD after 1 week	>1000	Not detected

**Table 3:** Effect of hypromellose (HPMC) content on particle sizes of silymarin (SLM) suspensions prepared by PureNano^TM^ and their freeze-dried (FD) samples.

Compound	Condition	Ratio (*w/w*)	Particle Size (nm)	PDI
		1/0.5	227.5	0.045
	PureNano^TM^	1/1	387.6	0.165
SLM/HPMC		1/3	1240.9	0.463
		1/0.5	>1000	0.115
	FD	1/1	617.0	0.221
		1/3	>1000	0.496

**Table 4:** Effect of cryoprotectants on particle sizes of silymarin/hypromellose nanoparticles (SLM/HPMC NPs) in the presence of various cryoprotectants.

Compound	Cryoprotectant	Ratio (*w/w/w*)	Particle Size (nm)	PDI
	Erythritol	1/1/1	253.8	0.108
	Mannitol	1/1/1	271.8	0.170
SLM/HPMC	Trehalose	1/1/1	265.2	0.160
	α-CyD	1/1/1	249.1	0.149
	HP-β-CyD	1/1/1	254.8	0.225

**Table 5:** Silymarin (SLM) contents in silymarin/hypromellose nanoparticles (SLM/HPMC NPs) in the presence of erythritol (Ery), α-cyclodextrins (α-CyD), and hydroxypropyl (HP)-β-CyD.

Sample	Content (%)	Theoretical Content (%)
SLM/HPMC/Ery FD	32.25 ± 0.01	
SLM/HPMC/α-CyD FD	31.30 ± 0.64	33.33
SLM/HPMC/HP-β-CyD FD	31.85 ± 0.41	

## Supplementary Materials

The following supporting information can be downloaded at: https://www.mdpi.com/article/10.3390/pharmaceutics14020394/s1, Figure S1: X-ray diffraction patterns of various samples.
