# Supplementary material for: Hydroxypropyl-β-cyclodextrin Enhances Oral Absorption of Silymarin Nanoparticles Prepared Using PureNano™ Continuous Crystallizer
Source: Pharmaceutics. 2022 Feb 10;14(2):394. doi: 10.3390/pharmaceutics14020394 (PMC8880042; doi:10.3390/pharmaceutics14020394)
Supplement: Supplementary file 1 [file pharmaceutics-14-00394-s001.zip › pharmaceutics-1591884-supplementary.pdf]

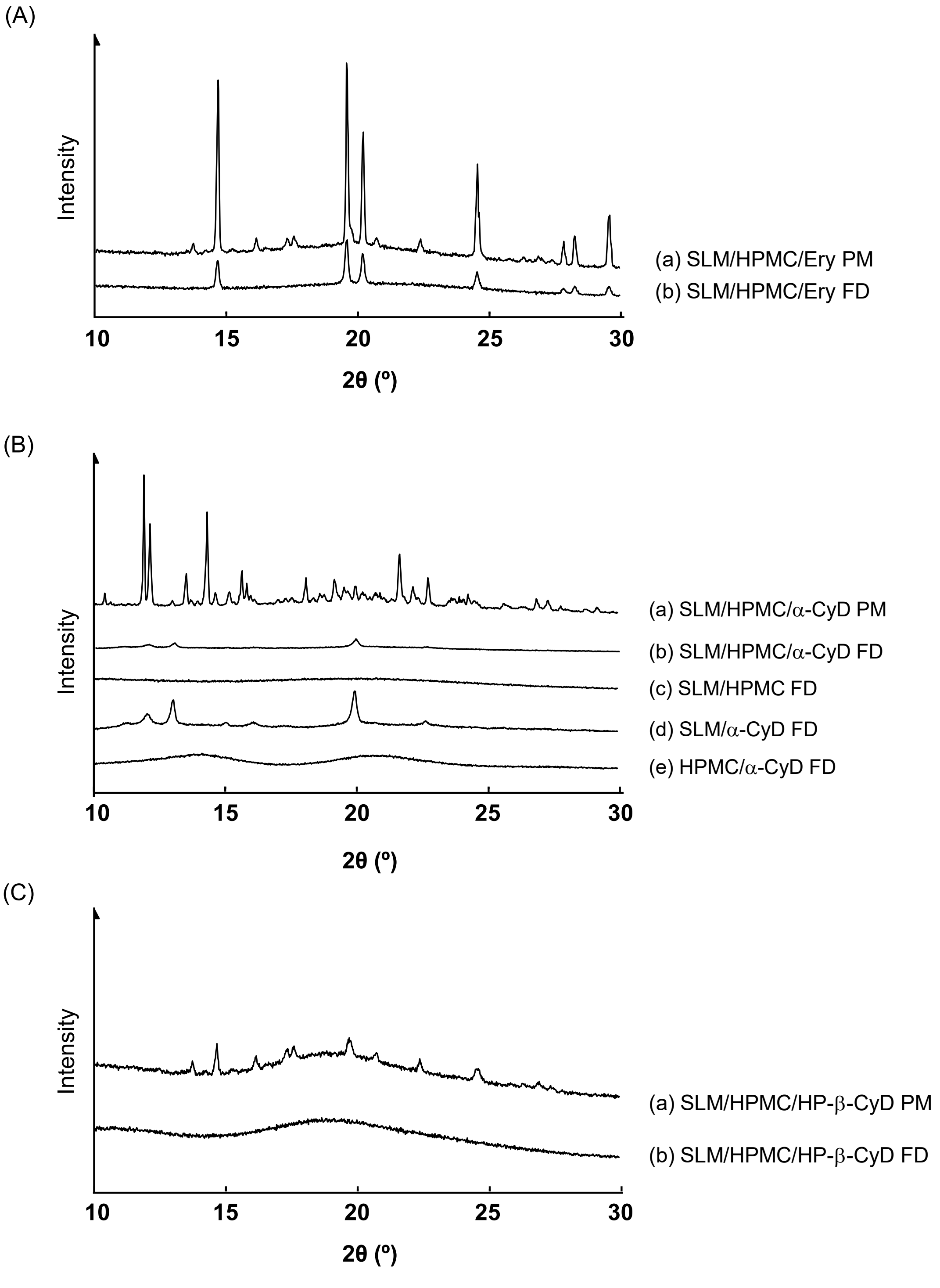

Supplemental Figure S1. X-ray diffraction patterns of various samples

The weight ratio of SLM/HPMC, SLM/α-CyD and HPMC/α-CyD was 1/1. The weight ratio of SLM/HPMC/Stabilizers was 1/1/1.
